# Targeted drug delivery system for ovarian cancer microenvironment: Improving the effects of immunotherapy

**DOI:** 10.3389/fimmu.2022.1035997

**Published:** 2022-11-03

**Authors:** Hongling Peng, Xiang He, Qiao Wang

**Affiliations:** Department of Gynecology and Obstetrics, Development and Related Diseases of Women and Children Key Laboratory of Sichuan Province, Key Laboratory of Birth Defects and Related Diseases of Women and Children, Ministry of Education, West China Second Hospital, Sichuan University, Chengdu, Sichuan, China

**Keywords:** ovarian cancer, TME (tumor microenvironment), drug delivery system (DDS), immunotherapy, chemotherapy

## Abstract

Immunotherapies have shown modest benefits in the current clinical trials for ovarian cancer. The tumor microenvironment (TME) in an immunosuppressive phenotype contributes to this “failure” of immunotherapy in ovarian cancer. Many stromal cell types in the TME (e.g., tumor-associated macrophages and fibroblasts) have been identified as having plasticity in pro- and antitumor activities and are responsible for suppressing the antitumor immune response. Thus, the TME is an extremely valuable target for adjuvant interventions to improve the effects of immunotherapy. The current strategies targeting the TME include: 1) eliminating immunosuppressive cells or transforming them into immunostimulatory phenotypes and 2) inhibiting their immunosuppressive or pro-tumor production. Most of the effective agents used in the above strategies are genetic materials (e.g., cDNA, mRNA, or miRNA), proteins, or other small molecules (e.g., peptides), which are limited in their target and instability. Various formulations of drug delivery system (DDS) have been designed to realize the controlled release and targeting delivery of these agents to the tumor sites. Nanoparticles and liposomes are the most frequently exploited materials. Based on current evidence from preclinical and clinical studies, the future of the DDS is promising in cancer immunotherapy since the combination of agents with a DDS has shown increased efficacy and decreased toxicities compared with free agents. In the future, more efforts are needed to further identify the hallmarks and biomarkers in the ovarian TME, which is crucial for the development of more effective, safe, and personalized DDSs.

## Introduction

Ovarian cancer is the leading cause of gynecological cancer-associated death (1). Epithelial ovarian cancer, especially high-grade serous ovarian carcinoma, is the most common histologic subtype. Most patients newly diagnosed with ovarian cancer can benefit from the conventional first-line treatment that mainly consists of debulking surgery and platinum-based chemotherapy. However, due to difficulties in the early detection of this disease, the majority of patients with ovarian cancer are initially diagnosed at the advanced stage (most frequently with extrapelvic peritoneal metastasis), which is known for its high recurrence rate and poor prognosis. Although first recurrences are frequently sensitive to chemotherapy, patients with recurrent disease will eventually face the problem of chemotherapy resistance. Thus, novel adjuvant therapies, such as targeted therapy and immunotherapy, are needed in order to provide new therapeutic opportunities for these patients. The use of certain targeted therapies, such as anti-angiogenic agents and poly(ADP-ribose) polymerase inhibitors (PARPi), have been approved by the US Food and Drug Administration (FDA) for patients with advanced-stage or recurrent ovarian cancer either in combination with chemotherapy or in maintenance monotherapy. In contrast, no immunotherapeutic agents have been approved by the FDA in ovarian cancer.

The immunotherapeutic strategies currently investigated in clinical trials for ovarian cancer include: 1) immune modulators, such as immune checkpoint inhibitors (ICIs) and immune regulatory cytokines; 2) cancer vaccines (e.g., dendritic cell vaccination); and 3) chimeric antigen receptor-modified T (CAR-T) cell therapy, as a representative variant of adoptive cell therapies (ACTs) (2–9). Data from important clinical trials on these therapies were reviewed (**Table 1**). Despite the rapid development of immunotherapies in basic research, the immunotherapy response rates among ovarian cancer patients remain modest, as shown by these clinical trials. The tumor microenvironment (TME) is considered a vital factor in the antitumor efficacy of immunotherapies (10). The TME refers to an intricate ecosystem of different immune cells, endothelial cells (ECs), stromal cells, and the extracellular matrix (ECM), as well as their networking interactions with tumor cells (11). The TME plays an important role in cancer development, progression, and metastasis (12). A drug delivery system (DDS), defined as a formulation or a device that enables a therapeutic substance to selectively reach its site of action, can enhance the efficacy and reduce the side effects of drugs, which makes it a promising strategy to improve the effects of cancer immunotherapy by targeting the TME (13). In this review, we discussed the strategies to improve the efficacy of immunotherapy in ovarian cancer with DDS, especially for those targeting the TME.

**Table 1:** Clinical trials of immunotherapy in ovarian cancer.

Immunotherapy	ID	Phase	*N*	Drugs	Conclusion	Reference
ICI	NCT02580058 JAVELIN 200	III	361	1) Avelumab; 2) Avelumab + PLD; 3) PLD	No benefit	(2)
	NCT03038100 IMagyn050	III	1,300	1) Atezolizumab + PC + bevacizumab; 2) Placebo + PC + bevacizumab	No benefit	(3)
	NCT02718417 JAVELIN 100	III	988	1) PC; 2) PC + avelumab, avelumab maintenance; 3) PC, avelumab maintenance	Terminated	–
	NCT02608684 PemCiGem	II	24	Pembrolizumab + standard treatment	No benefit	(4)
	NCT02811497	II	28	Durvalumab + DNA hypomethylating agent	No benefit	(5)
	NCT02865811	II	26	Pembrolizumab + PLD	Clinical benefit	(7)
	NCT02431559	II	40	Durvalumab + PLD	Clinical benefit	(6)
	NCT03899610	II	23	Durvalumab + tremelimumab + chemotherapy	Clinical benefit	(8)
ICI+PARPi+VEGFi	NCT03740165 KEYLYNK-001	III	1,086	1) Pembrolizumab + olaparib; 2) Pembrolizumab + placebo; 3) Placebo + PC + bevacizumab	Recruiting	–
ICI+PARPi+VEGFi	NCT03737643 DUO-O	III	1,056	1) Durvalumab + olaparib; 2) Durvalumab + placebo; 3) Placebo + PC + bevacizumab	Recruiting	–
CAR-T	NCT02498912	I	18	MUC16-CAR-T cells	Recruiting	–
	NCT02159716	I	19	MSLN CAR-T cells	Patients showed stable disease	–
	NCT03585764	I	18	FRαCAR-T cells	Recruiting	–
	NCT05225363	I	33	Tumor-associated glycoprotein 72 (TAG72) antigen CAR-T cells	Recruiting	–
	NCT03907527	I	71	PRGN-3005 UltraCAR-T cells (co-express a CAR-targeting MUC16 and IL-15)	Recruiting	–
Vaccine	NCT02764333	II	27	FRα vaccine (TPIV 200) + durvalumab	Clinical benefit	–
	NCT02346747	II	91	Gemogenovatucel-T vaccine (Vigil) + chemotherapy	Clinical benefit	(9)
	NCT00001827	II	21	P53 vaccine + IL2	Terminated	–

ICI, immune checkpoint inhibitor; PARPi, poly(ADP-ribose) polymerase inhibitors; VEGFi, vascular endothelial growth factor inhibitors; CAR-T, chimeric antigen receptor-modified T; PLD, pegylated liposomal doxorubicin; MSLN, mesothelin; PC, paclitaxel+carboplatin.

## Role of the TME in immunotherapy

In the TME, tumor cells coexist and interact with immune cells [e.g., macrophages, neutrophils, dendritic cells (DCs), natural killer (NK) cells, and lymphocytes] and non-immune cells (e.g., fibroblasts and ECs) (14). The TME is shaped by tumor cells to promote tumor development and to respond to stress, stimulation, and treatment. The total TME cannot be simply explained as a unitary “antitumor” or “pro-tumor” environment, but rather a dynamic and plastic system with characteristics such as hypoxia, nutrient deficiency, inflammation, immunosuppression, and angiogenesis. The patterns of the TME in solid tumors are tightly associated with the clinical outcomes of cancer patients (15, 16).

### Immune cells

Most solid tumors are infiltrated by myeloid and lymphoid lineage-derived immune cells within the TME playing significant roles in the antitumor response or tumor progression.

Tumor-associated macrophages (TAMs) are a major subpopulation of the myeloid lineage-derived cells in the ovarian TME playing critical roles in the crosstalk between the TME and tumor cells. TAMs are highly plastic, with two functional phenotypes. Depending on the TME, TAMs can differentiate into either the pro-inflammatory M1 macrophages with antitumor activity or the anti-inflammatory M2 macrophages with pro-tumor activity. M1 macrophages possess cytotoxicity and stimulate immunity. In ovarian cancer, TAMs are predominantly M2 macrophages, secreting immunosuppressive cytokines and taking part in regulating T cells, remodeling the ECM, and angiogenesis (17).

Neutrophils are of the myeloid lineage cells and comprise the major subpopulation among polymorphonuclear leukocytes, representing the first line of innate immunity against pathogens. The detection of neutrophils within the TME is an indirect parameter of cancer-related inflammation. Tumor-associated neutrophils can exert antitumor (N1 phenotype) or pro-tumor (N2 phenotype) functions, depending on the related stimulating factors and cytokines within the TME.

Myeloid-derived suppressor cells (MDSCs) are a heterogeneous population of immature myeloid cells that differ in morphology and function from terminally differentiated mature myeloid cells (e.g., macrophages, neutrophils, and DCs). When activated and accumulating in peripheral lymphoid tissues and the tumors, they are implicated in suppressing immunity and promoting tumor progression. The different function and differentiation of MDSCs are related to the different phenotype of the TME (18).

DCs, well known as the most powerful or professional antigen-presenting cells (APCs), are crucial in immune responses and represent the “bridge” between the innate and adaptive immune systems (19). There are both myeloid and lymphoid DCs. After capturing antigens, DCs process them and present peptides to T cells *via* the major histocompatibility complex (MHC), subsequently initiating a series of T-cell activity. Analogous to TAMs, tumor-infiltrating DCs are of plasticity. They can be immunogenic or tolerogenic depending on the TME. DEC205^+^CD11c^+^MHC-II^low^ immature DCs act on tumor vascularization and immunosuppression. The performance of DCs varies at different stages of tumor development (20). As shown in mouse models of ovarian cancer, tumor growth was prevented by infiltrating DCs at the early stage. However, at the advanced stage, immunosuppressive phenotypes of DCs were found in the TME (21).

NK cells are innate lymphoid cells and effector cells of the innate immune system. These cells do not rely on human leukocyte antigen (HLA)-mediated recognition of neoantigens. The expressed receptors (such as CD16, NKG2D, and natural cytotoxicity receptor) on NK cells mediate the killing of tumor cells (22). NK cells also exert effects on the adaptive immune response to cancer through secreting inflammatory cytokines. Defects in NK cell function, such as aberrant receptor expression or inability to effectively secrete cytotoxic molecules, are possible mechanisms of tumor immune escape (23).

Lymphocytes are important components of the TME. B lymphocytes can mediate innate immunity, secrete antibodies, and act as professional APCs. Within the TME, both the pro- and antitumor activities of B lymphocytes have been identified in solid tumors as different subsets playing diverse roles. T lymphocytes are pivotal in adaptive immunity. CD4^+^ and CD8^+^ T cells are mature T cells in the TME (24). After antigen presentation, T cells are activated and start to differentiate into various effector subsets. CD4^+^ T cells perform a wide variety of functions and are best known as T helper (Th) cells, including Th1, Th2, and Th17, and regulatory T cells (Tregs). Tregs inhibit the activation of immune response and are crucial in the mechanism of tumor immune escape. CD8^+^ T cells, known as cytotoxic T lymphocytes (CTLs), work by specifically recognizing and killing tumor cells (25). Besides CTLs, gamma-delta (γδ) T lymphocytes can kill ovarian cancer cells when activated by positive signals. There are several activating receptors (e.g., NKG2D) and inhibitory receptors that regulate γδ T-cell killing. The presence of tumor-infiltrating lymphocytes (TILs) has been reported as a positive prognostic factor in a number of solid cancers, including ovarian cancer (26–29).

### Non-immune cells

Cancer-associated fibroblasts (CAFs) are an important type of stromal cells in the TME and produce various components in the ECM. Normal fibroblasts can prevent the emergence of neoplastic lesions and inhibit tumorigenesis. CAFs, on the contrary, play a role in immune suppression and angiogenesis, showing pro-tumor function (30). Malfunctioning blood vessels and excessive ECM within the TME impair blood flow and limit the delivery of oxygen, nutrients, and antibodies and immune cells. This results in hypoxia and low pH and induces the production of molecules with immunosuppressive activities, such as vascular endothelial growth factor (VEGF). Angiogenesis, which refers to the formation of new blood capillaries from preexisting vasculature, generating the tumor-associated neovasculature, addresses the need to transport nutrients and oxygen, as well as metabolic wastes and carbon dioxide, in the TME (31). This creates a vicious cycle in which angiogenesis can induce immunosuppression in the TME, while certain suppressive immune cells can induce angiogenesis (32). ECs are the cells lining the vessels within the TME, which play an important role in angiogenesis.

### Immunosuppressive modulators

Transforming growth factor-beta (TGF-β) is one of the most important immunosuppressive cytokines. TGF-β proteins are produced by many cell types, including all white blood cell lineages, in a latent form. Activated TGF-β complexes with other factors and binds to TGF-β receptors, physiologically maintaining immunological self-tolerance and suppressing cancer. However, within the TME, aberrant TGF-β activation and signaling promote tumor progression by stimulating epithelial–mesenchymal transition, angiogenesis, CAF activation, and immunosuppression (33). TGF-β also regulates the generation and functions of many immune cell types, including promoting the expansion of Tregs and inducing the polarization of the pro-tumor N2 phenotype of neutrophils (34).

Immune checkpoint molecules are inhibitory receptors that are expressed on immune cells, negatively regulating immune response in the TME. Cytotoxic T lymphocyte antigen-4 (CTLA-4) and programmed cell death protein-1 (PD-1) are the two checkpoint inhibitors garnering the most attention. CTLA-4 is a negative regulator of T cells that counteracts with the co-stimulatory molecule CD28. PD-1 is expressed by T cells and binds to one of the two ligands [programmed death-ligand 1 (PD-L1) and PD-L2] that are expressed on tumor and immune cells (16, 35). The PD-1/PD-L1 pathway is an important axis for restricting tumor immunity.

### Phenotype of cancer-immune TME

**Figure 1:**
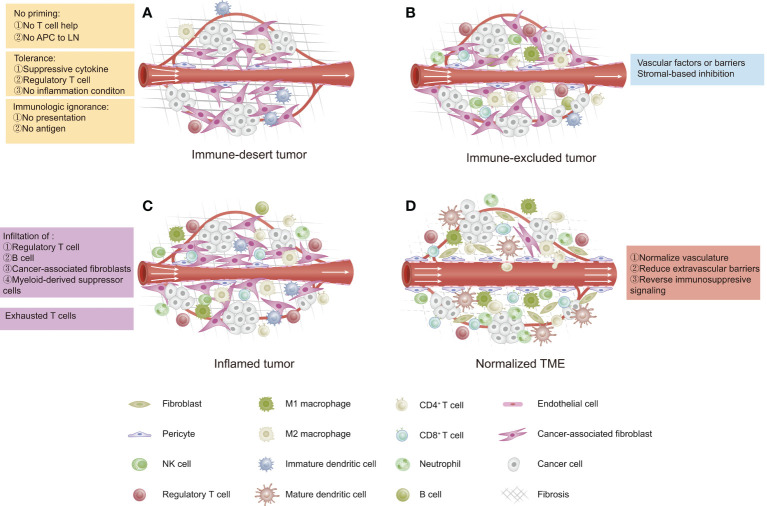
Three phenotypes of cancer immunity in the tumor microenvironment (TME). **(A)** Immune-desert type: characterized by a lack of antitumor immune cells due to low immunogenicity. **(B)** Immune-excluded type: characterized by immune cells restricted at the tumor periphery due to tumor vascular barriers and stromal-based inhibition. **(C)** Inflamed type: characterized by immune cells infiltrating the tumor parenchyma and expressing pro-inflammatory cytokines, but a failed antitumor immune response. **(D)** Normalized TME by reversing immunosuppressive signaling, improving tumor perfusion, and reducing barriers.

The cancer–immunity cycle mainly consists of the following processes: 1) release and presentation of tumor-associated antigens (TAAs); 2) priming and activation of T cells; 3) trafficking of T cells to tumors; 4) infiltration of T cells into tumors; and 5) recognition and killing of tumor cells by T cells (36). The cancer-immune TME in solid tumors has been categorized as “hot” (high immunogenicity) or “cold” (low immunogenicity), which mainly depends on the status of immune cell infiltration within the tumor space. This difference in the cancer-immune phenotype of the TME suggests that hot tumors exhibit stronger responses to immunotherapy than do “cold” tumors (37). The cancer-immune TME can be categorized into three main phenotypes (**Figure 1**) (13): 1) immune-desert type, which shows low immunoactivity due to immunological ignorance (lack of neoantigens), the induction of tolerance, or a lack of appropriate T-cell priming or activation. Tumors of this phenotype are the least responsive to ICIs; 2) immune-excluded type, which is characterized by immune cell trafficking in the tumor periphery due to a limited chemokine state or the barriers of vessels, stroma, and ECM. Tumors of this phenotype are potentially more sensitive to ICIs than those of the immune-desert phenotype; 3) inflamed type, which refers to a dysfunctional antitumor immune response with the infiltration of a number of immune cells (including Tregs, MDSCs, suppressive B cells, and CAFs). CD8^+^ CTLs are dysfunctional and exhausted. Tumors of this phenotype have the most sensitivity to ICIs.

In most cases, ovarian cancer is considered as a cold tumor and has an immune-desert TME with a low immune cell density either inside or outside of the tumor (38), which is not likely to trigger a strong immune response or respond to immunotherapy. Thus, in order to improve the effects of immunotherapy in ovarian cancer, new strategies are needed to “normalize” the antitumor immunity within the ovarian TME, for example, strategies that target the tumor vasculature, the extravascular barriers, the immunosuppressive status, and the cancer–immunity cycle (13).

## Role of the DDS in immunotherapy

A DDS is a carrier of a therapeutic substance designed to control its release, improve its solubility and stability, overcome the biological barriers, and target the site. The processes of a DDS include the administration of the therapeutic substance, the release of the active ingredients, and the subsequent transport of the active ingredients to the site of action (39–41). Various materials (organic or inorganic) such as lipids, glycans, and proteins, as well as synthetic polymers, have been utilized for the development and improvement of the DDS (42). According to the particle size, the DDS can be further categorized into nano-, micro-, or macroscale (43). Here, we focused on the DDS at the nanoscale (nanocarrier), which is designed and developed based on the application of nanotechnology (44). Nanocarriers, acknowledged to have enhanced permeability and retention (EPR) effect, help deliver chemotherapeutic or immunotherapeutic drugs selectively to tumors, which results in increased efficacy and reduced systemic toxicity of drugs (45, 46). A wide variety of platforms have been investigated as nanocarriers in preclinical and clinical research, including lipid-based (liposomes), polymer-based (polymeric micelles, dendrimers, and polymeric nanoparticles), drug-conjugated (antibody–drug conjugates), and viral and inorganic nanoparticles (47, 48).

As described above, the clinical use of immunotherapy in many solid cancers is confronted with difficulties related to efficacy and challenges related to safety. With regard to safety, serious adverse effects such as autoimmunity and nonspecific inflammation limit the broad implementation of immunotherapy. For example, systemically administered pro-inflammatory cytokines can lead to autoimmune toxicities and even result in a “cytokine storm.” Thus, a DDS can be utilized to provide safer and more effective cancer immunotherapies (49).

### DDS for immune modulators

When it comes to the immune modulatory agents, the DDS can improve the pharmacokinetics and biodistributions of the cytokines and ICIs. Conjugating polyethylene glycol (PEG) has been clinically tried to improve the half-life and stability of pro-inflammatory cytokines (50). In order to reduce the toxicity associated with the systemic administration of drugs, binding cytokines to liposomes or collagen-binding domains can enable the selective delivery of drugs to tumors and draining lymph nodes (51, 52). Matrix-binding molecular conjugates were designed to bind the ICIs to the tumor (53). With this intratumoral and peritumoral delivery, these conjugates remain more localized in the TME than the unmodified ICIs.

### DDS for cancer vaccines

With regard to cancer vaccines, a DDS can protect tumor antigens from degradation and enable intracellular delivery (49, 54). For example, lipid-based formulations were designed to improve the instability and inefficient delivery of messenger RNA (mRNA), which were shown to be efficacious in preclinical animal models and in initial clinical studies (55, 56). Furthermore, drug conjugates are utilized to improve the effect of subunit vaccines (such as peptides) in combination with molecular adjuvants by targeting DCs in the lymph nodes. The accumulation of these conjugates in the lymph nodes resulted in increased T-cell priming, improved antitumor efficacy, and reduced systemic toxicity in animal models (57). Other platforms such nanoparticles and dendrimers are also being investigated as carriers in cancer vaccines (58, 59).

### DDS for ACTs

A major challenge for ACTs in solid cancers is the localization of T cells at disease sites. Biomaterial-based DDSs, such as polymeric scaffolds, have been investigated to solve this issue (60). Polymeric scaffolds coated with collagen-mimetic peptides bind antigen-specific T cells and deliver them locally within the TME (61). Another challenge for ACTs is that the viability and function of the transplanted cells rapidly decline after administration. High dosages of adjuvant drugs are required to maximize the efficacy of ACTs. T-cell-conjugated nanoparticles, in which an immune-stimulating DDS is conjugated directly to the surface of T cells, were designed to improve the efficacy (62, 63). DDSs activating T cells *in vivo* were also designed, which offered another alternative to conventional ACTs (62). As an example, synthetic/artificial APCs composed of lipids or polymers and functionalized with antigens and surface ligands were designed to mimic APCs in order to activate T cells (64, 65).

### DDS for combination therapy

Cancer combination therapy is a promising approach to improving antitumor efficiency and has been investigated in preclinical and clinical studies (66). DDSs can also be exploited in cancer combination treatments and in modulating the immunogenicity in the TME, especially for immunotherapeutic strategies for cold tumors. Tumor cells undergoing selective chemotherapy and radiation can release signals that enhance immunogenicity and induce the activation of T cells locally or systematically, which has been reported to induce immunogenic cell death (ICD) (67). Apart from ICD, chemotherapy is also helpful in normalizing the TME by increasing perfusion and alleviating hypoxia (68). Thus, the combination of chemotherapy and immunotherapy can provide a synergistic effect in antitumor treatment. In this combination therapy, DDS helps achieve the delivery of sustained drug concentrations to enhance the therapeutic effects and reduce the side effects (69). As an example of this combination effect, liposomal DDSs were complexed with PD-L1-blocking signals to form nanoparticles that are targeted to tumor tissue (70). Mice bearing colorectal tumors were injected with both these nanoparticles and the chemotherapy drug (oxaliplatin). The results suggested that oxaliplatin may induce cold tumors to turn into hot tumors, subsequently making them susceptible to immunotherapy, exhibiting reduced toxicity. As another example, twin-like core–shell nanoparticles were developed for synchronous biodistribution and a separate cell targeting delivery of sorafenib (an antiangiogenic agent) and IMD-0354 (a TAM re-polarization agent) to cancer cells and TAMs, respectively, to promote superior synergistic antitumor efficacy and M2 macrophage polarization ability (71). Liposome- and micelle-based chemoimmunotherapies were also designed and studied in animal models (72–74).

## DDS targeting the TME in ovarian cancer

**Figure 2:**
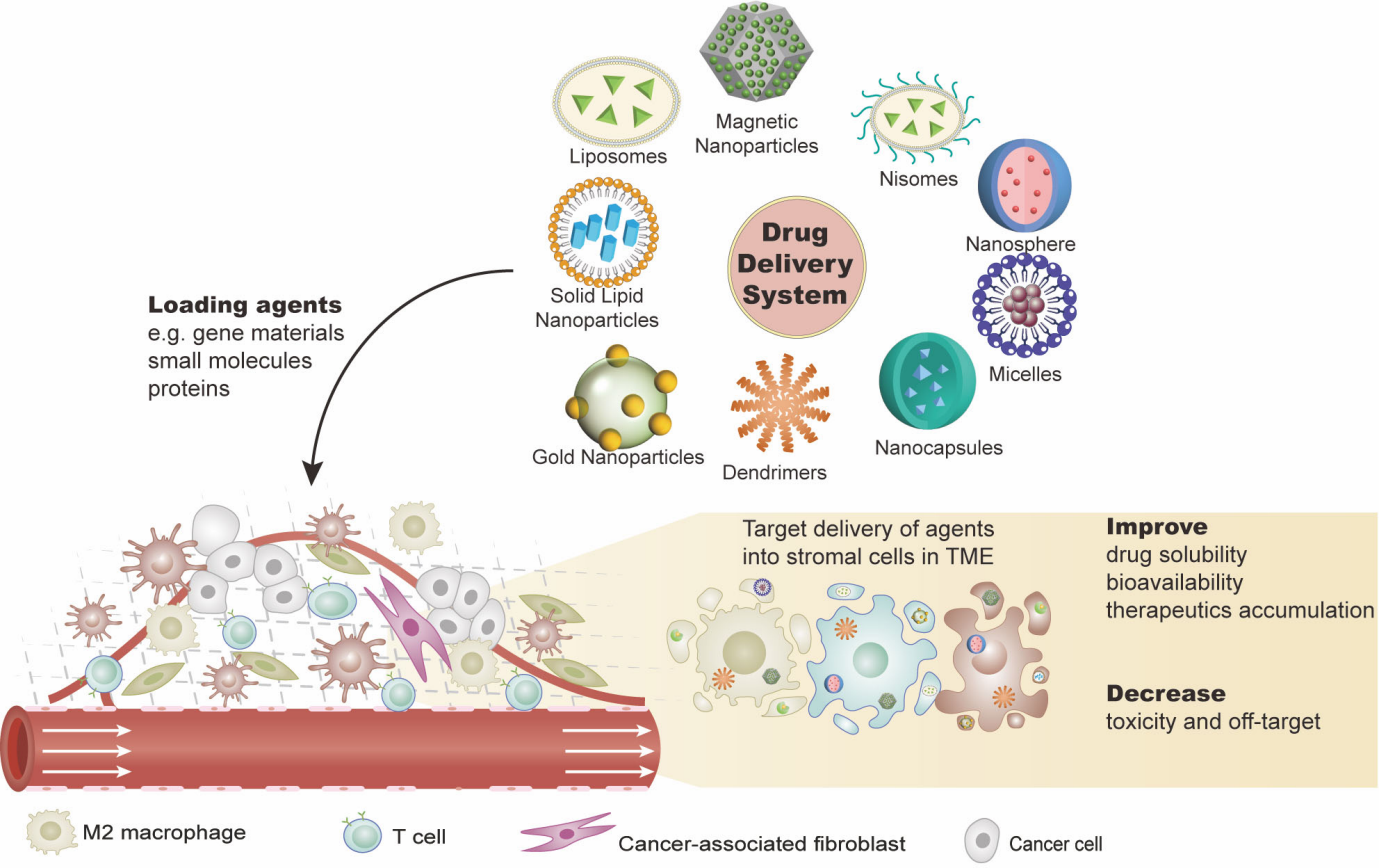
Drug delivery systems (DDSs) targeting the ovarian tumor microenvironment (TME). DDSs carry effective agents such as gene materials (e.g., cDNA, mRNA, or miRNA), proteins, or other small molecules (e.g., peptides) into the tumor sites. These agents are expected to work on: 1) eliminating the immunosuppressive cells or transforming them into immunostimulatory phenotypes and 2) inhibiting the immunosuppressive or pro-tumor production of the stromal cell. This combination of agents with DDS not only improves the solubility and stability of the agents but also fulfills the target delivery with reduced toxicities. There are various DDS platforms, such as liposomes and nanoparticles.

A lot of effort has been made to develop new strategies for improving the antitumor efficacy of immunotherapy for ovarian cancer. As described above, the TME in ovarian cancer shows low immunogenicity, which is an obstacle to immunotherapy. The application of a DDS targeting the TME in ovarian cancer has been explored in preclinical and early clinical studies (**Table 2**, **Figure 2**).

**Table 2:** Drug delivery systems (DDSs) currently developed to target the tumor microenvironment (TME) in ovarian cancer.

Target in the TME	Delivery technology	Effective agents	Combined therapy	Study design	Reference
**TAMs (CD47/SIRPα signaling pathway)**	Virus	Therapeutic genes	None	Preclinical study	(75, 76)
**TAMs (Toll-like receptor)**	Liposomes	Resiquimod	PD-1 blockade	Preclinical study	(77)
**TAMs (repolarization)**	Nanoparticles	MicroRNA-125b	Intraperitoneal paclitaxel	Preclinical study	(78)
**TAMs (repolarization)**	Nanoparticles	IRF5 mRNA	None	Preclinical study	(79)
**M1 macrophages**	Nanotubes	Doxorubicin	None	Preclinical study	(80)
**DCs**	Nanoparticles	Small interfering RNA	None	Preclinical study	(81, 82)
**γδ T cells**	Liposomes	Aminobisphosphonates	ACTs	Preclinical study	(83)
**CAFs and MDSCs**	Nanoparticles	Therapeutic genes	None	Preclinical study	(84, 85)
**Low immunogenicity**	Virus	Peptides	PD-1 blockade	Preclinical study	(86)
**Low immunogenicity**	Nanoparticles	IL-6	PD-1 blockade	Preclinical study	(87)
**Low immunogenicity**	Liposomes	Doxorubicin	PD-1 blockade	Early-phase clinical study	(6, 7)

TAMs, tumor-associated macrophages; DCs, dendritic cells; CAFs, cancer-associated fibroblasts; MDSCs, myeloid-derived suppressor cells; ACTs, adoptive cell therapies.

### DDS targeting immune cells

Generally, an increased immune cell infiltration is associated with better prognosis in ovarian cancer. TAMs, as major components within the ovarian TME and playing critical roles in various stages of tumor progression, represent a promising target for cancer drug delivery (88, 89). Signal regulatory protein α (SIRPα) is the surface ligand of CD47 on TAMs. CD47/SIRPα signaling plays an important role in tumor immune escape (75). In a previous study, a virus was used to carry therapeutic genes that blocked the CD47/SIRPα signaling pathway in ovarian cancer. This effectively increased macrophage infiltration into the tumor and enhanced tumor cell killing. Similar to CD47, the CD24 in tumor cells binds the inhibitory receptor on the surface of TAMs to promote the immune escape of ovarian cancer cells. Ovarian cancer with a decreased CD24 expression was found to be more sensitive to CD47 blockers, indicating co-targeting CD24 and CD47 as a candidate for cancer immunotherapy (76).

In particular, in line with the distinct functions of the two different phenotypes, a high number of classically activated macrophages (M1 macrophages) in the ovarian TME is closely correlated with better prognosis, while increased M2 macrophage infiltration is correlated with poor prognosis (90, 91). Clodronate-loaded liposomes are effective tools for macrophage ablation. Long-term usage of thymoquinone was reported to increase the infiltration of M2 macrophages in the ascites in models of ovarian cancer. When clodronate liposomes were used in combination with thymoquinone, the number of TAMs was significantly reduced while the proportion of M2 macrophages was increased, resulting in the promotion of tumor growth. Toll-like receptor (TLR) 7/TLR8 agonists are potent immunostimulatory molecules that repolarize TAMs. However, these small molecules have poor pharmacokinetic profiles and carry the risk of inducing severe systemic toxicity, which limits their administration *via* intratumoral injection. Anionic liposomes were used to deliver TLR agonists (e.g., resiquimod) administered intraperitoneally in ovarian cancer-bearing mice (77). The results showed the promotion of M1 macrophage polarization and T-cell infiltration in the TME. In addition, the percentage of Tregs was reduced in the TME. These liposome-formulated TLR agonists could also enhance the efficacy of PD-1 blockade. Furthermore, other DDSs were also designed to be administered intraperitoneally. Certain relatively large anionic nanoparticles (>100 nm) have been shown to be able to selectively accumulate in TAMs in a mouse model of metastatic ovarian cancer, while other particles that were smaller than 100 nm, or cationic, or administered intravenously did not show TAM targeting (92). This ability of these nanoparticles opens the possibility of targeting the TAMs in ovarian cancer. Another hyaluronic acid-based nanocarrier encapsulating MiR-125b, a microRNA affecting the phenotype polarization of TAMs, was designed. These nanoparticles specifically targeted TAMs in the peritoneal cavity and repolarized them to the immune-activating phenotype in an ovarian cancer mouse model. Furthermore, these nanoparticles, when combined with intraperitoneal paclitaxel, enhanced the antitumor efficacy of paclitaxel without inducing systemic toxicity (78). Another study using a mouse model of ovarian cancer explored a nanocarrier that could deliver *in vitro*-transcribed mRNA encoding M1-polarizing transcription factors to reprogram TAMs. The infusion of *IRF5* mRNA and IκB kinase beta (IKKβ) nanoparticles reversed the immunosuppressive state of the TAMs by reprograming M2 macrophages into M1 macrophages (79).

Macrophages can also act as carriers themselves. In a mouse model of intraperitoneally metastatic ovarian cancer, engineered doxorubicin-loaded M1 macrophages were designed to transfer drug cargoes into tumor cells *via* a tunneling nanotube pathway. These engineered macrophages were found to penetrate into and accumulate deep within disseminated tumor lesions, resulting in the elimination of metastatic tumors and increase in survival (80).

Immature DCs and MDSCs have been identified as responsible for suppressing the antitumor immune response. These cancer-associated immune cells within the ovarian TME emerge as alternative therapeutic targets complementing current immunotherapies (49). DDSs carrying gene materials or small molecules were engineered to eliminate these cancer-associated immune cells and to transform them into an immunostimulatory phenotype. For instance, linear polyethylenimine-based nanoparticles encapsulating small interfering RNA (siRNA) were described and could be selectively engulfed by tumor-resident DCs when injected into the peritoneal cavity of ovarian cancer-bearing mice (81). These nanoparticles induced the activation of DCs. Another basic research indicated that ovarian cancer-associated DCs are also capable of engulfing liposomes carrying plasmid DNA-encoding cytokines in order to support the activities of CTLs (82).

The DDS was also exploited in targeting and modulating lymphocytes. The efficacy of ACT using γδ T cells could be enhanced by aminobisphosphonates such as alendronic acid, the clinical exploitation of which was limited by the inefficient and nonselective uptake of these agents in tumor cells. Aminobisphosphonates were encapsulated within liposomes and investigated in a preclinical study. The results showed that the liposomal alendronic acid rendered advanced tumors susceptible to γδ T-cell-mediated shrinkage and was proven markedly superior when compared with free drug delivered intravenously (83).

### DDS targeting non-immune cells

The biological mechanism of CAFs suggests that CAFs represent a therapeutic target in cancer immunotherapy. The current interventions on CAFs mainly include: 1) inhibiting the pro-tumor signaling pathway between CAFs and other stromal cells to reverse tumorigenesis, angiogenesis, and immunosuppression in the TME and 2) inhibiting the production of ECM by CAFs to reduce solid pressure in the TME. For example, fibroblast activation protein (FAP) is a specific marker for CAFs. In a preclinical study of ovarian cancer, upon delivering FAP siRNA to CAFs, the growth of tumor cells was inhibited, with a decreased level of CAFs (84, 85). Many other therapeutic agents such as mRNA and small molecules are good mediators for CAF modulation. Other DDSs, such as a lipid-coated calcium phosphate and lipid–protamine–DNA nanoparticles, were developed as delivery platforms targeting CAFs and have been studied in animal models of pancreatic and bladder cancer (93).

### DDS targeting immune modulators

There are various pieces of preclinical evidence that the DDS could exhibit prolonged tumor residence and favorable intratumoral distribution of immune modulators. As one example, cowpea mosaic virus (CPMV) combined with an anti-PD-1 peptide (SNTSESF) was examined as an alternative to the expensive antibody therapies using ICIs. This combination resulted in the increased efficacy of anti-PD-1 peptides in a mouse model of intraperitoneal ovarian cancer. Moreover, an increased potency against metastatic ovarian cancer was only observed when SNTSESF was conjugated to CPMV, but not when given as a free peptide (86). As another example, the hyperactivation of interleukin 6 (IL-6) is a hallmark in the TME of ovarian cancer progression. The effect of IL-6 is achieved *via* activating several signaling pathways such as the RAS–RAF–MAPK and AKT–PI3K–mTORC1 pathways. Dual inhibitor-loaded nanotherapeutics (DiLNs) that can co-deliver PI3K and MAPK inhibitors were also developed. In *in vitro* studies, DiLNs were shown to be stable for over a month and released the drugs in a sustained manner. *In vivo* studies showed that the combination of DiLNs with an anti PD-L1 antibody resulted in superior antitumor effect and longer survival (87).

Pegylated liposomal doxorubicin (PLD) is the first FDA-approved cancer nanomedicine and a paradigm of DDS utilized in ovarian cancer. Besides its use in chemotherapy, PLD can also contribute positive immunomodulatory efforts due to the anthracyline-induced translocation of calreticulin to the cell surface, the upregulation of MHC-I and Fas surface expression, and ICD (94). The efficacy of anti-PD-1 therapy plus PLD has been demonstrated in the early stages of clinical studies. A single-arm, multicenter phase II trial of ovarian cancer indicated that the combination of pembrolizumab (an anti-PD-1 antibody) and PLD was manageable, without unexpected toxicities, and showed preliminary evidence of a clinical benefit. The response rate and survival in this study were both higher than historical comparisons of PLD alone or anti-PD-1 agents alone (7). A similar result was shown in a phase I/II study of durvalumab (an anti-PD-LI antibody) combined with PLD for platinum-resistant recurrent ovarian cancer (6). More clinical trials (e.g., NCT02839707) are ongoing.

Controlled neoantigen release is a major challenge for successful immunotherapy, especially in tumors of the immune-desert phenotype such as ovarian cancer. Many TAAs in solid tumors are not confined to tumor tissues but can also be found in normal somatic tissues, which results in off-target toxicities. Tumor-specific antigens are good candidates for targeting and localizing to the tumor sites in immunotherapy, such as NY-ESO-1 (a cancer–testis antigen). The expression of NY-ESO-1 is restricted in normal somatic tissues, concomitant with a re-expression in solid epithelial cancers (95, 96). NY-ESO-1 vaccines have been designed and investigated in preclinical studies and early phase trials. In ovarian cancer, combination therapies of the NY-ESO-1 vaccine, PLD, and decitabine in 10 patients with recurrent disease showed promising results. Six of the 10 patients had disease stabilization or partial clinical response (97). HLA-A2-restricted peptides presented by tumor cells are candidate antigens for the development of a therapeutic cancer vaccine. A novel liposomal platform called DepoVax™ (DPX; Halifax, NS, Canada) was used to enhance the potency of the HLA-A2-restricted peptide vaccine (DPX-0907). The phase I clinical trial of DPX-0907 exhibited a 61% immune response rate (98). There are many other formulations designed based on the low immunogenicity of TAAs in ovarian cancer, such as a slow-release dendrime of cowpea mosaic virus for *in situ* vaccine delivery (99).

## Conclusions and perspectives

The immunosuppressive TME with low immunogenicity is a big obstacle in the implementation of immunotherapy for solid tumors such as ovarian cancer (15, 100, 101). It is believed that a TME-targeted strategy is a valuable adjuvant therapy for ovarian cancer. Given the complexity of the interaction network in the TME, there remains the challenging task of developing drugs or therapies simultaneously targeting multiple pathways. The combined administration of two or more targeted therapeutics, or even the addition of immunotherapeutics and chemotherapeutics, is expected to exhibit a synergistic antitumor effect and improve each other’s efficacy. However, toxicity is a major concern.

The application of DDSs in immunotherapy is mainly based on the advantage of selective accumulation in tumor sites relative to normal tissues, which greatly reduces the risk of toxicities. In addition, the peritoneal metastasis and ascites in ovarian cancer make the DDS a potentially valuable approach to carry the load since abundant peritoneal phagocytes can engulf the carriers and accumulate the load inside the tumors, acting as Trojan horses. Various DDS-based strategies have been designed and examined in preclinical studies. Based on the evidence from previous research works, we consider the future of DDSs, especially for nanocarriers, as promising in the immunotherapy for ovarian cancer, not only as a direct delivery platform of immunotherapeutic agents but also as a carrier of genes or functional molecules that can transform the immunosuppressive TME into an immunostimulatory TME. However, not all basic research can result in clinical treatment for patients. In addition to the manufacturing technique and costs, there will be many more concerns when it comes to clinical translation. Furthermore, there is limited information on the long-term biosafety and bioeffect of the component materials themselves in these carriers.

More efforts are needed to further understand the TME in ovarian cancer in order to identify more specific hallmarks and biomarkers that will help in the design and development of more DDSs with better effectivity and biosafety, or even for personalized therapy.

## Data availability statement

The original contributions presented in the study are included in the article/supplementary material. Further inquiries can be directed to the corresponding author.

## Author contributions

QW and HP performed the literature search and screening. HP drafted the manuscript. QW and XH reviewed and revised the draft. All authors contributed to the article and approved the submitted version.

## Funding

This study was supported by the Department of Science and Technology of Sichuan Province (2022NSFSC1307).

## Conflict of interest

The authors declare that the research was conducted in the absence of any commercial or financial relationships that could be construed as a potential conflict of interest.

## Publisher’s note

All claims expressed in this article are solely those of the authors and do not necessarily represent those of their affiliated organizations, or those of the publisher, the editors and the reviewers. Any product that may be evaluated in this article, or claim that may be made by its manufacturer, is not guaranteed or endorsed by the publisher.

## Abbreviations

ICIs, immune checkpoint inhibitors; ACTs, adoptive cell therapies; TME, tumor microenvironment; DDS, delivery systems; PARPi, poly(ADP-ribose) polymerase inhibitor; FDA, Food and Drug Administration; CAR-T, chimeric antigen receptor-modified T; NKs, natural killer cells; DCs, dendritic cells; TGF-β, transforming growth factor beta; TAMs, tumor-associated macrophages; MDSCs, myeloid-derived suppressor cells; APCs, antigen-presenting cells; MHC, major histocompatibility complex; CTLs, cytotoxic lymphocytes; CAFs, cancer-associated fibroblasts; CTLA-4, cytotoxic T lymphocyte antigen-4; PD-1, programmed cell death protein-1; TAAs, tumor-associated antigens; ECM, extracellular matrix; EPR, enhanced permeability and retention; PEG, polyethylene glycol; PLD, pegylated liposomal doxorubicin; TLR, Toll-like receptor; IL, interleukin.
